# Implementation of a biochemical, clinical, and genetic screening programme for familial hypercholesterolemia in 26 centres in Spain: The ARIAN study

**DOI:** 10.3389/fgene.2022.971651

**Published:** 2022-08-29

**Authors:** Teresa Arrobas Velilla, Ángel Brea, Pedro Valdivielso

**Affiliations:** ^1^ Laboratorio de Nutrición y Riesgo Cardiovascular de Bioquímica Clínica, Unidad de Lípidos, Hospital Universitario Virgen de la Macarena, Sevilla, Spain; ^2^ Servicio de Medicina Interna, Unidad de Lípidos, Hospital de San Pedro, Logroño, España; ^3^ Servicio de Medicina Interna, Unidad de Lípidos, Hospital Virgen de la Victoria, Instituto de Investigación Biomédica de Málaga (IBIMA), Universidad de Málaga, Málaga, Spain

**Keywords:** familial hypercholesterolemia (FH), clinical laboratory, lipid units, Dutch lipid clinic network criteria, screening

## Abstract

**Background:** Familial hypercholesterolemia (FH) is clearly underdiagnosed and undertreated. The aim of this present study is to assess the benefits of FH screening through a joint national program implemented between clinical laboratories and lipid units.

**Methods:** All clinical laboratory tests from 1 January 2017 to 31 December 2018 were reviewed, and those with LDL cholesterol (LDL-C) levels >250 mg/dl were identified in subjects >18 years of age of both sexes. Once secondary causes had been ruled out, the treating physician was contacted and advised to refer the patient to an LU to perform the Dutch Lipid Clinic Network score and to request genetic testing if the score was ≥6 points. Next Generation Sequencing was used to analyse the promoter and coding DNA sequences of four genes associated with FH (*LDLR, APOB, PCSK9, APOE*) and two genes that have a clinical overlap with FH characteristics (*LDLRAP1* and *LIPA*). A polygenic risk score based on 12 variants was also obtained.

**Results:** Of the 3,827,513 patients analyzed in 26 centers, 6,765 had LDL-C levels >250 mg/dl. Having ruled out secondary causes and known cases of FH, 3,015 subjects were included, although only 1,205 treating physicians could be contacted. 635 patients were referred to an LU and genetic testing was requested for 153 of them. This resulted in a finding of sixty-seven pathogenic variants for FH, 66 in the *LDLR* gene and one in *APOB*. The polygenic risk score was found higher in those who had no pathogenic variant compared to those with a pathogenic variant.

**Conclusion:** Despite its limitations, systematic collaboration between clinical laboratories and lipid units allows for the identification of large numbers of patients with a phenotypic or genetic diagnosis of FH, which will reduce their vascular risk. This activity should be part of the clinical routine.

## Introduction

FH is a monogenic disease characterised by the presence of high levels of LDL cholesterol particles (LDL-C) from birth which, if not properly treated, is associated with premature coronary artery disease and mortality. The disease is caused by pathogenic variants in the LDL receptor (*LDLR*), apolipoprotein B (*APOB*) and proprotein convertase subtilisin/kexin type 9 (*PCSK9*) genes. A phenotypic diagnosis can be obtained using various scoring systems such as the Dutch Lipid Clinic Network (DLCN) score (Mostaza et al., 2022), which considers the LDL-C levels of patients, the levels of first-degree relatives, the presence or absence of xanthomas or corneal arcus in the affected subject, and the personal or family history of cardiovascular disease (CVD). Diagnostic confirmation requires the sequencing of candidate genes and identification of the corresponding pathogenic variant (Brown et al., 2020).

The prevalence of FH is unknown, although it was initially estimated that the disease affected one in 500 people. Two recent meta-analyses of 44 and 42 studies give a prevalence of one in 313 (Beheshti et al., 2020) and one in 311 (Hu et al., 2020), respectively. In addition, a recent study in our country suggest an even higher prevalence among patients treated in health care centres in the Catalonia area with estimates of one in every 189 people being affected (Zamora et al., 2017). It is widely believed that FH is frequently underdiagnosed and undertreated (Nordestgaard et al., 2013).

The identification of patients with FH allows the initiation of lipid-lowering treatment consisting mainly of statins in combination or not with other drugs, such as ezetimibe or PCSK9 inhibitors (PCSK9i). Prompt initiation of treatment for FH can drastically reduce the development of CVD and the risk of mortality (Versmissen et al., 2009; Luirink et al., 2019).

Universal (Groselj et al., 2018) and cascade screening programes (Leonardi-Bee et al., 2021), which allows early diagnosis especially in children (Groselj et al., 2022) now exist in some countries and around the world (Wilemon et al., 2020). Opportunistic testing is also possible for patients who have survived an acute coronary syndrome (Mortensen et al., 2016; Amor-Salamanca et al., 2017; Dyrbuś et al., 2019). Over the last decade, considerable interest has been shown in the identification of patients with FH by using analytical records from community laboratories (Bell et al., 2014; Kirke et al., 2015; Mirzaee et al., 2019). There are limited data in Spain on the use and benefits of centralised analytical data screening in testing for FH (Gutiérrez-Cortizo et al., 2021; Sabatel-Pérez et al., 2021).

The objective of the ARIAN study is to assess the benefits of FH screening through the implementation of a joint national programme between clinical laboratories and lipid units.

## Materials and methods

The design of the ARIAN study has already been published (Arrobas Velilla et al., 2021). To summarise, fifty-five clinical laboratories from different Spanish hospitals, which perform biochemical analyses in their respective primary care referral areas, were invited to participate.

In 2019, a retrospective identification of patients aged ≥18 years with a direct or calculated LDL-C > 250 mg/dl (>6.7 mmol/L) and a blood triglyceride concentration <400 mg/dl during the period of 1 January 2017 to 31 December 2018 was obtained through a consultation of the laboratory information system (LIS) of each centre. After reviewing their clinical history, patients were excluded if they had secondary hyperlipidemia (pregnancy, cholestasis, non-substituted hypothyroidism, nephrotic syndrome, among others.) or if they had been previously diagnosed with FH.

Each clinical laboratory contacted the physician requesting the analysis to inform them that their patient was suspected of having FH and to recommend that they be referred to the lipid unit (LU) of the health care centre. In no case was the patient contacted directly. At the LU, the score obtained by the DLCN method was assessed and in cases where the score was ≥6, genetic testing was advised. Each centre had funding for five genetic tests.

Whole blood or saliva samples for deoxyribonucleic acid (DNA) analysis were collected from patients who were accepted into the study. The genetic study was performed using the LIPID inCode^®^ *in vitro* diagnostic platform (GENinCode Plc, Oxford, United Kingdom). This platform analyses the promoter and coding DNA sequences as well as exon-intron boundaries (±25 bp) of four genes associated with FH (*LDLR, APOB, PCSK9, APOE*) and two genes associated with other conditions that have a partial clinical overlap with FH characteristics [autosomal recessive hypercholesterolemia (*LDLRAP1*) and lysosomal acid lipase deficiency (*LIPA*)] by Next Generation Sequencing (NGS) (Amor-Salamanca et al., 2017). The test has been optimised to detect heterozygous, homozygous, and hemizygous single nucleotide variants (SNVs) and small insertions/deletions (indels) (<15 bp) in the six analysed genes, as well as copy number variants (CNVs) in the *LDLR* gene. All detected variants with possible clinical relevance (pathogenic, likely pathogenic or variants of unknown significance) were confirmed by Sanger sequencing or MLPA analysis, depending on the variant type.

Variant data analysis is described in the supplementary material. Variants with a minor allele frequency <1% in the general population were considered non common variants. The potential pathogenicity of rare variants was evaluated by considering the recommendations of the American College of Medical Genetics and Genomics (ACMG) and the Clinical Genome Resource (ClinGen) Familial Hypercholesterolemia consensus guidelines for *LDLR* variant classification (Richards et al., 2015; Chora et al., 2022), by which different criteria are evaluated: type and variant frequency, functional data if available, scientific support, and computational information for predicted pathogenicity in genomic (PolyPhen2, Provean v.1.1.3, MutationTaster2 and REVEL) or intronic (MaxEntScan, NNSplice, FSPLICE, and GeneSplicer) regions, among others (Richards et al., 2015). Moreover, information on >2,200 FH-related genomic variants included in a private database was also considered to complete the evaluation of genetic variants. Variants with clinical relevance were reported as pathogenic, likely pathogenic, and variants of unknown significance (VUS).

In the case of polygenic hypercholesterolemia, the variants included in the LDL-C Score (rs7412, rs429358, rs1367117, rs4299376, rs629301, rs1564348, rs1800562, rs3757354, rs11220462, rs8017377, rs6511720, and rs2479409) were also analysed by NGS and according to Talmud et al. (Talmud et al., 2013).

### Statistical analysis

Data are shown as n (%) or mean ± SD. To compare between groups, we used the χ^2^ test for qualitative variables and the ANOVA test for continuous variables. A *p*-value of <0.05 was considered significant. Data were analysed with SPSS 25.0 (IBM).

### Ethical aspects

The study obtained authorisation from the ethics committee of the Virgen de la Macarena Hospital, Seville, on the 16th February 2019. The participating clinical laboratories also obtained approval from the ethics committees of their centres. The study protocol conforms to the ethical guidelines of the 1975 Declaration of Helsinki and all patients gave written informed consent to the genetic test and their clinical data.

## Results

**FIGURE 1:**
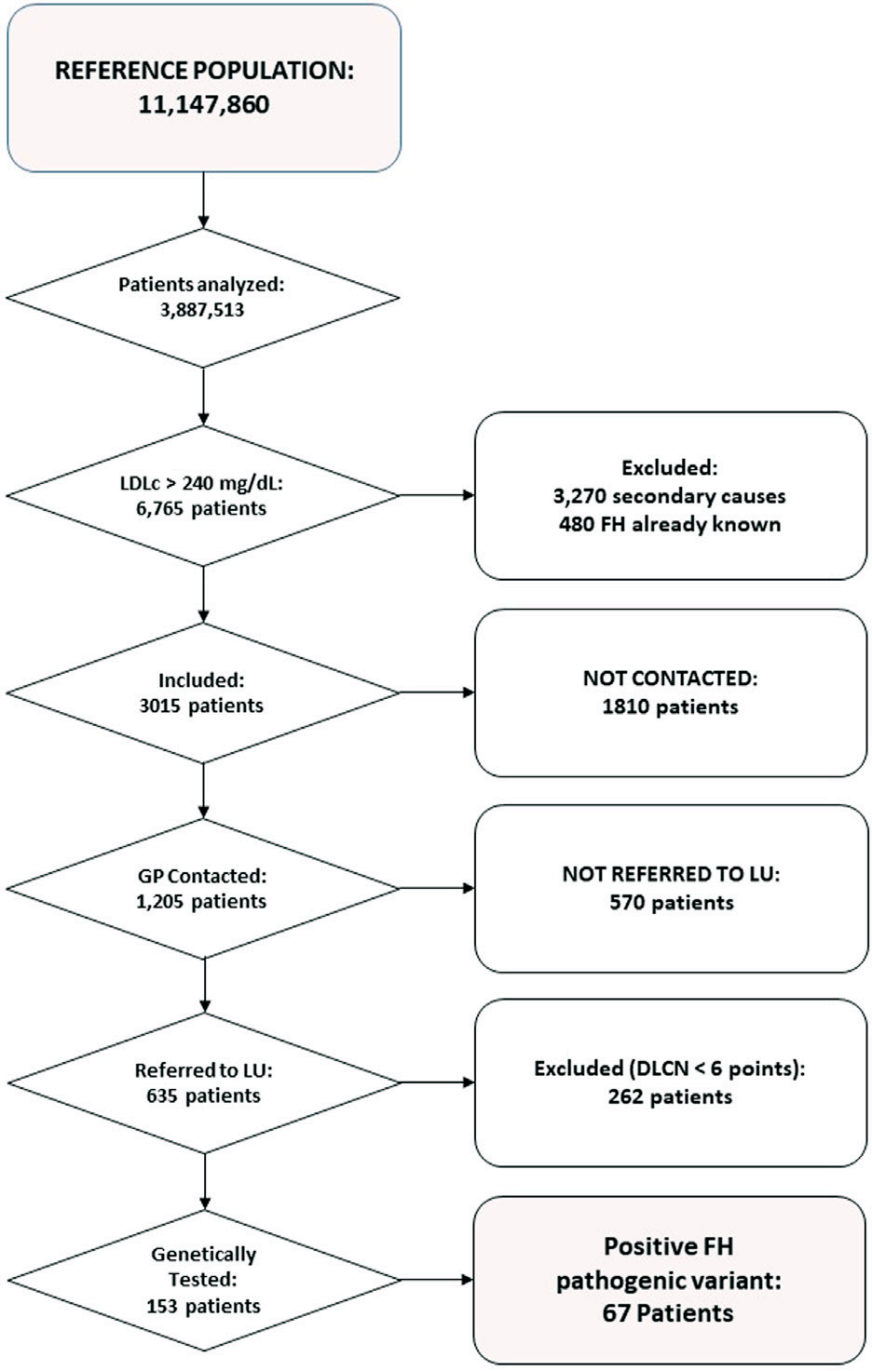
Flow-chart of the study, showing patients and analyses at each step.

In the period analysed, twenty-six (45%) centres actively participated in the study, with a reference population in their health areas of 11,147,860 individuals. Serum samples from 3,887,513 adult patients (≥18 years) were analysed, of whom 6,765 had LDL-C levels above 250 mg/dl. Secondary causes that could account for hypercholesterolemia were found in 3,270 patients; 480 patients were already known to have FH (317 had been diagnosed by DLCN and a further 163 by genetic confirmation). Thus, the final sample was 3,015 patients. Of these, only 1,205 patients could be contacted and among this group, 635 (52%) visited their LU. Then, among these patients, 373 obtained a DLCN score ≥6. A genetic test was requested for 153 patients, and a pathogenic variant for FH was found in sixty-seven (45%) of them. No one patient showed a pathogenic variant at *APOE* gene. Figure 1 summarises these data in a flow chart.

Table 1 summarises the general characteristics of the sample, indicating that younger patients in whom a pathogenic variant was found exhibited higher DLCN scores and higher LDL-C than the rest of the participants. Polygenic risk score was significantly higher in subjects with genetic test negative or inconclusive compared to those with a pathogenic variant (*p* < 0.05, ANOVA test).

**TABLE 1:** Demographic and clinical data from patients genetically tested for Familial Hypercholesterolemia.

		Genetic analyses			
	All 153 (100%)	Negative 67 (42%)	Inconclusive 19 (13%)	Positive 67 (45%)	
Age (years-old)	52 ± 15	57 ± 9	53 ± 17	46 ± 17	<0.05
Sex (men)	48 (31%)	21 (32%)	7 (29%)	22 (31%)	NS
LDLc (mg/dl)	265 ± 96	256 ± 108	247 ± 91	280 ± 88	NS
PRS	1.97 0.18	1.01 ± 0.19	0.97 ± 0.15	0.92 ± 0.20	<0.05
DLCN score					<0.05
Probable	97 (63%)	51 (48%)	15 (80%)	32 (33%)	
Certainty (>8)	56 (37%)	16 (25%)	4 (20%)	36 (66%)	

Data are shown as mean ± SD, or n (%). LDLc: LDL, cholesterol. PRS: Polygenic Risk Score. DLCN: dutch lipid clinic network.

Table 2 summarizes the studies published on screening for FH using centralized laboratory data and a comparison with the present study. Rates of positive genetic testing ranges from 46 to 68%, mostly depending on the inclusion criteria.

**TABLE 2:** Screening for Familial Hypercholesterolemia using centralized laboratory data.

Author	Location and duration	Patients analyzed	LDLc cut-off point	DLCN	Genetic test (positives/solicited)
Scicali et al. (2018)	Catania (Italy) 48 months	1,575	>190 mg/dl& first degree child relatives with cLDL >160 mg/dl	Possibles + probables 56	26/56 (46%)
Gutierrez-Cortizo (2021)	Huelva (Spain) 36 months	37,400	>200 mg/dl	654 possibles	81/152 (53%)
				129 probables	
Sabatel Perez et al. (2021)	Toledo (Spain) 48 months	Unreported	>220 mg/dl	285 possibles	57/84 (68%)
				84 probables	
Arrobas-Velilla et al. (2021) (present study)	National (Spain) 24 months	3,887,513	>250 mg/dl	262 possibles	69/153 (45%)
				373 probables	

DLCN: ducth lipid clinic network score.

Table 1 in the supplementary material presents the variants found in the patients analysed. No homozygous variants were found in the *LDLRAP1* and *LIPA* genes.

## Discussion

Our study confirms that systematic screening of LDL-C levels in the clinical laboratory and collaboration with the lipid unit allows for the identification of patients with FH phenotype and its genetic confirmation. This should lead to a better degree of lipid control and improved prevention of cardiovascular disease.

It is noteworthy that 3,270 among 6,765 patients with an LDL-C higher than 250 mg/dl were considered as secondary hypercholesterolemia. However, a recent study showed that the prevalence of secondary causes increase with LDL-C levels, reaching the 52% among those with LDL-C > 300 mg/dl (Jasani et al., 2022).

Despite widespread knowledge of genetic testing and increased accessibility, FH continues to be diagnosed at a late stage and early diagnosis is one of the main forms in reducing the clinical impact of the disease (Vallejo-Vaz et al., 2021). There is a clear need to incorporate new methods with which to identify patients with FH, such as genomic screening in populations or during blood donation, biochemical analysis at birth or during the period of childhood immunisation, and the development of algorithms derived from machine learning (Bradley et al., 2022). A recent study in Australia considered genomic screening of populations aged 18–40 years to be cost-effective from a healthcare system perspective if the cost of the test was less than 160 (Marquina et al., 2021).

As was expected in our study, the frequency of pathogenic variants gradually increased from probable FH to definite FH according to the Dutch Lipid Clinic Network criteria (Taranto et al., 2021) as 66% of the cases with a monogenic diagnosis had a DLCN score of certainty (>8) (see Table 1). In our study, only 67 out of 153 (46%) patients were found to have a pathogenic variant causing FH, a figure which is similar to that published by Scicali et al. (Scicali et al., 2018). However, in this paper, patients with LDL-C > 190 mg/dl and a DLCN score ≥4 were included. Conversely, this rate of finding pathogenic variants is slightly lower than other series of a similar design published in Spain, which range from 53 to 68% (Gutiérrez-Cortizo et al., 2021; Sabatel-Pérez et al., 2021) (see Table 2).

It is noteworthy that no pathogenic variant was found after genetic analysis in sixty-five (42%) patients in our study with LDL-C levels >250 mg/dl, and VUS variants were found in 19 (13%) patients. This highlights the importance of polygenic risk in severe primary hypercholesterolemia (Futema et al., 2018). As expected, patients with a negative or inconclusive genetic study (VUS) had a significantly higher polygenic score than in patients who were shown to have a pathogenic variant (Talmud et al., 2013) (Table 2). Although PRS is associated with increased LDL-C levels and risk of coronary heart disease, knowledge of PRS does not add value to risk calculation if incorporated into a model that includes age, sex and LDL-C levels (Tromp et al., 2021). Although severe polygenic hypercholesterolaemia does not present the same acute risk of cardiovascular disease as monogenic forms, the risk is still high (Khera and Hegele, 2020) and some authors propose family cascade screening using LDL-C as a guide (Jarauta et al., 2020).

Although the study design (Arrobas Velilla et al., 2021) allowed the identification of patients with severe hypercholesterolemia (3,015) and among them sixty-seven carriers of pathogenic FH variants, we believe the performance of the study leaves room for improvement. Having previously ruled out secondary causes and known cases of FH, the primary care physician could be contacted regarding only 1,205 (40%) of the aforementioned 3,015 individuals with LDL-C levels >250 mg/dl. Geographical dispersion among hundreds of primary care centres participants in the study and lack of personal phone number or email in the ordering test form, may have contributed to the low rate of contacting. Moreover, among those who could be contacted, only 635 (53%) were referred to the LUs. Although these findings are discouraging, they are in line with studies of a similar design to ours. Of 4,517 subjects with LDL-C levels >170 mg/dl identified by a general laboratory, only 597 (13%) agreed to be clinically evaluated for suspected FH. Of these, thirty were genetically tested and eight showed a pathogenic variant in the gene responsible for FH (Kirke et al., 2015). In another Australian study of 100 cases with LDL-C > 250 mg/dl detected in a clinical laboratory, there was a 27% referral rate to a LU when primary care physicians were informed of suspected FH cases and the high risk of CV disease and instructed to refer their patients to a LU. Furthermore, ninety-six individuals in the control group exhibited the same LDL-C levels; however, their physicians were not notified thus contributing to an even lower referral rate (4%) (Bell et al., 2014). Considering these data as a whole, there is much room for improvement in PC regarding the recognition of FH as an extremely high-risk vascular condition. At the same time, there is evidence of a certain “resistance” to refer cases to LUs, where sometimes complex diagnosis and treatment of these patients can be carried out, as many require combined treatments including PCSK9i, which is a class of prescription drugs dispensed in hospitals (Ascaso et al., 2019; Visseren et al., 2021).

Finally, it is striking that among the 6,765 patients identified in clinical laboratories with LDL-C levels >250 mg/dl, 480 already had a previous clinical or genetic diagnosis of FH (Figure 1). This finding clearly reveals undertreatment and is particularly striking considering that in Spain, FH patients benefit from a very low co-payment (reduced contribution) (Boletín Oficial del Estado, 2003), paying only 4 euros for statins and ezetimibe (regardless of type and dose), and are entitled to a full reimbursement for PCSK9i.

Among the limitations, it is worth mentioning that genetic studies were financed for only five cases per centre since many centres do not have their own means to carry out genetic studies. Furthermore, there was a low response rate to refer patients to an LU by those PC physicians who could be contacted. Other limitation of our study has been to include only adult population; that means that we have lost the opportunity to identify children with FH and, as a consequence, to delay the diagnosis in such group of patient with the highest chance to prevent atherosclerosis earlier.

## Conclusion

The systematic review by a clinical laboratory of cases with extremely high LDL-C levels, communication of these to primary care physicians, and assessment of cases by lipid units allows for the identification of many patients with a phenotypic and/or genetic diagnosis of FH. This clinical activity should be part of routine clinical practice.

## Data Availability

The raw data supporting the conclusions of this article will be made available by the authors, Data are available from the authors upon reasonable request and with permission of Spanish Arteriosclerosis Society.
