# Supplementary material for: Implementation of a biochemical, clinical, and genetic screening programme for familial hypercholesterolemia in 26 centres in Spain: The ARIAN study
Source: Front Genet. 2022 Aug 29;13:971651. doi: 10.3389/fgene.2022.971651 (PMC9465084; doi:10.3389/fgene.2022.971651)
Supplement: Supplementary file 1 [file Table1.DOCX]

**Supplementary Material**

**Table 1. Variants found as Likely pathogenic**

| ***Gene*** | **rs** | **gDNA** | **cDNA** | **Exon** | **Protein** |
| --- | --- | --- | --- | --- | --- |
| *LDLR (4)* | rs879254375 | g.11200090C>G | c.-135C>G | 1 | . |
| *LDLR* | . | g.11213338A>T | c.191-2A>T | 2i | . |
| *LDLR* | rs730882078 | g.11213390C>T | c.241C>T | 3 | p.(Arg81Cys) |
| *LDLR* | rs121908025 | g.11213408T>C | c.259T>C | 3 | p.(Trp87Arg) |
| *LDLR* | rs1555803439 | g.11216254_11216268del | c.672_686del | 4 | p.(Asp224_Glu228del) |
| *LDLR* | rs879254693 | g.11218100T>G | c.850T>G | 6 | p.(Cys284Gly) |
| *LDLR* | rs140241383 | g.11218108C>A | c.858C>A | 6 | p.(Ser286Arg) |
| *LDLR* | rs879254728 | g.11218187T>C | c.937T>C | 6 | p.(Cys313Arg) |
| *LDLR* | . | g.11221448_11221450del | c.1060+1_1060+3del | 7i | . |
| *LDLR* | rs879254801 | g.11222259G>A | c.1130G>A | 8 | p.(Cys377Tyr) |
| *LDLR* | rs879254879 | g.11224213C>A | c.1361C>A | 10 | p.(Thr454Asn) |
| *LDLR* | rs879254989 | g.11226881_11226887delinsGCCCAAT | c.1698_1704delinsGCCCAAT | 11 | p.(Ile566_Leu568delinsMetProAsn) |
| *LDLR* | rs185098634 | g.11227549C>T | c.1720C>T | 12 | p.(Arg574Cys) |
| *LDLR* | rs875989930 | g.11227574T>C | c.1745T>C | 12 | p.(Leu582Pro) |
| *LDLR* | rs879255012 | g.11227583T>A | c.1754T>A | 12 | p.(Ile585Asn) |
| *LDLR* | rs758493597 | g.11238765A>G | c.2389+4A>G | 16i | . |

**Variants found as Pathogenic**

| ***Gene*** | **rs** | **gDNA** | **cDNA** | **Exon** | **Protein** |
| --- | --- | --- | --- | --- | --- |
| *LDLR* | . | g.(11234021_11238683)_(11238762_11240188) | c.(2311+1_2312-1)_(2389+1_2390-1)del | Del ex. 16 | *LDLR* |
| *LDLR* | . | g.(11213463_11215895)_(11218191_11221327)del | c.(313+1_314-1)_(940+1_941-1)del | Del ex. 4-6 | . |
| *LDLR* |  | g.(11216277_11217240)_(11224439_11226769) | c.(694+1_695-1)_(1586+1_1587-1)del | Del ex. 5-10 | . |
| *LDLR (6)* | . | g.(11222316_11223953)_(11227675_11230767)del | c.(1186+1_1187-1)_(1845+1_1846-1)del | Del ex. 9-12 | . |
| *LDLR* | rs201016593 | g.11200235G>A | c.11G>A | 1 | p.(Trp4Ter) |
| *LDLR* | rs879254453 | g.11213410_11213411delinsAG | c.261_262delinsAG | 3 | p.(Trp87Ter) |
| *LDLR (2)* | rs112029328 | g.11213463G>C | c.313+1G>C | 3i | . |
| *LDLR* | rs875989897 | g.11213464dup | c.313+2dup | 3i | . |
| *LDLR* | rs879254482 | g.11215928T>C | c.346T>C | 4 | p.(Cys116Arg) |
| *LDLR* | rs879254536 | g.11216046G>A | c.464G>A | 4 | p.(Cys155Tyr) |
| *LDLR (4)* | rs121908026 | g.11216112C>T | c.530C>T | 4 | p.(Ser177Leu) |
| *LDLR* | rs376459828 | g.11216172G>A | c.590G>A | 4 | p.(Cys197Tyr) |
| *LDLR* | rs771917370 | g.11216213C>T | c.631C>T | 4 | p.(His211Tyr) |
| *LDLR* | rs730882096 | g.11221414G>A | c.1027G>A | 7 | p.(Gly343Ser) |
| *LDLR* | rs769737896 | g.11221435C>T |  | 7 | p.(Arg350Ter) |
| *LDLR (4)* | rs879254811 | g.11222297A>T | c.1168A>T | 8 | p.(Lys390Ter) |
| *LDLR* | rs570942190 | g.11224013C>T | c.1246C>T | 9 | p.(Arg416Trp) |
| *LDLR (2)* | rs879254871 | g.11224109C>T | c.1342C>T | 9 | p.(Gln448Ter) |
| *LDLR* | rs775924858 | g.11224126G>A | c.1358+1G>A | 9i | . |
| *LDLR* | rs28942080 | g.11224419G>A | c.1567G>A | 10 | p.(Val523Met) |
| *LDLR (2)* | . | g.11226765_11226800del | c.1587-5_1617del | 10i-11 | . |
| *LDLR* | rs28941776 | g.11226829G>A | c.1646G>A | 11 | p.(Gly549Asp) |
| *LDLR (2)* | rs397509365 | g.11226873A>C | c.1690A>C | 11 | p.(Asn564His) |
| *LDLR* | . | g.11227348_11227553del | c.1706-187_1724del | 11i-12 | . |
| *LDLR (3)* | rs137929307 | g.11227604G>A | c.1775G>A | 12 | p.(Gly592Glu) |
| *LDLR (2)* | rs879255049 | g.11227675G>C | c.1845+1G>C | 12i | . |
| *LDLR* | rs879255056 | g.11230781G>A | c.1859G>A | 13 | p.(Trp620Ter) |
| *LDLR (2)* | rs746118995 | g.11230819C>T | c.1897C>T | 13 | p.(Arg633Cys) |
| *LDLR* | rs875989944 | g.11240196_11240204del | c.2397_2405del | 17 | p.(Val800_Leu802del) |
| *LDLR* | rs374045590 | g.11240274C>A | c.2475C>A | 17 | p.(Asn825Lys) |
| *APOB* | rs5742904 | g.21229160C>T | c.10580G>A | 26 | p.(Arg3527Gln) |

(N) below the gene indicates the numbers the variant has been found

**Variants of Unknown Significance**

| ***Gene*** | **rs** | **gDNA** | **cDNA** | **Exon** | **Protein** |
| --- | --- | --- | --- | --- | --- |
| *LDLR* | . | g.11221355G>T | c.968G>T | 7 | p.(Gly323Val) |
| *LDLR* | rs747507019 | g.11221366C>T | c.979C>T | 7 | p.(His327Tyr) |
| *LDLR* | rs879254810 | g.11222287C>A | c.1158C>A | 8 | p.(Asp386Glu) |
| *LDLR* | rs775657243 | g.11224208C>T | c.1359-3C>T | 9i | . |
| *LDLR* | rs72658865 | g.11227645G>A | c.1816G>A | 12 | p.(Ala606Thr) |
| *LDLR* | . | g.11238756C>T | c.2384C>T | 16 | p.(Pro795Leu) |
| *APOB* | rs145655926 | g.21228171C>T | c.11569G>A | 26 | p.(Val3857Met) |
| *APOB* | rs146377316 | g.21230565G>A | c.9175C>T | 26 | p.(Arg3059Cys) |
| *APOB* | . | g.21233352T>C | c.6388A>G | 26 | p.(Asn2130Asp) |
| *APOB* | rs914459572 | g.21245824C>A | c.2695G>T | 18 | p.(Val899Phe) |
| *APOB* | rs942258196 | g.21249785T>C | c.2119A>G | 15 | p.(Lys707Glu) |
| *APOB* | . | g.21250950A>G | c.1830-13T>C | 13i | . |
| *APOB* | . | g.21252502G>A | c.1617+9C>T | 12i | . |
| *APOB* | . | g.21266947C>A | c.-130G>T | . | . |
| *PCSK9* | rs747072726 | g.55509621C>T | c.313C>T | 2 | p.(Arg105Trp) |
| *PCSK9* | rs28385712 | g.55522980del | c.997-23del | 6i | . |
