# Supplementary material for: Implementation of a biochemical, clinical, and genetic screening programme for familial hypercholesterolemia in 26 centres in Spain: The ARIAN study
Source: Front Genet. 2022 Aug 29;13:971651. doi: 10.3389/fgene.2022.971651 (PMC9465084; doi:10.3389/fgene.2022.971651)
Supplement: Supplementary file 2 [file DataSheet1.DOCX]

## Appendix A. List of participating centres and researchers

**Hospital Virgen Macarena** (Sevilla): Begon˜a Gallardo Alguacil, Ramon Pérez Temprano, Mar Martínez Quesada, Miguel Ángel Rico. **Hospital Virgen del Rocío** (Sevilla): Lourdes Diez Herrán and Ovidio Mun˜iz Grijalbo. **Hospital Universitario de Badajoz**: Puriﬁcación García Yun, Francisca Jiménez-Mena Villar and Francisco Morales Pérez. **Hospital Gregorio Maran˜ón** (Madrid): Olga González Albarrán, Mercedes Herranz Puebla, Carolina Puertas Robles. **Hospital Universitari Vall d’Hebron**: Silvia Campos Anguila and Joan Lima Ruiz. **Hospital Universitario Marqués de Valdecilla**. (Santander): Armando Raúl Guerra Ruiz and José Luis Hernández Hernández. **Hospital Universitario Virgen de las Nieves and Hospital Universitario Clínico San Celicio** (Granada): José Vicente García Lario, Pablo González Busto, Fernando Rodríguez Alemán, María Mar Águila García, Fernando Jaén Ávila. **Hospital General de Valencia**: Goitzane Marcaida Benito and Juan José Tamarit Gracia. **Hospital Universitario Son Espasses** (Palma de Mallorca): Cristina Gómez Cobos and Juan Ramón Urgeles Planella. **Hospital Clínico Universitario Lozano Blesa** (Zaragoza): Luis Irigoyen Cucalón, José Antonio Gimeno Orna, José Ruiz Budría. **Hospital Juan Ramón Jiménez** (Huelva): Ignacio Vázquez Rico and Jessica Roa Garrido. **Hospital Universitario de Burgos**: Enrique Ruiz Pérez, María Maravi Álvarez, Laura de la Maza Pereg, María Victoria Poncela García, María Martin Palencia. **Hospital Virgen del Puerto** (Plasencia): David Pen˜alver Talavera, Montan˜a Jiménez Álvaro. **Hospital Universitario Infanta Cristina** de Parla (Madrid): Marco Puma Duque, Almudena Vigil Rodríguez, Juan Manuel Fernández Alonso. **Hospital Universitario de Gran Canaria Dr. Negrín**: José Alfredo Martin Armas, Magdalena León Mazorra, Casimira Domínguez Cabrera, Lidia Esther Ruiz Gracia. **Hospital Universitario San Jorge** (Huesca): José Puzo Foncillas. **Hospital Universitario de Bellvitge**: Xavier Pintó Sala and María José Castro Castro. **Hospital Miguel Servet** (Zaragoza): Fernando Civeira Murillo and Pilar Calmarza. **Hospital Insular de Gran Canaria**: Rosa Sánchez Hernández and Marta Rian˜o Ruiz. **Hospital Uni****versitario de Torrejón**: Camino García García-Lescun, María Almudena Amor, Eduardo Alegría. **Institut d’Assistencia Sanitaria Santa Caterina** (Gerona): Cristina Soler Ferrer and Mercé Montesino Costa. **Hospital San Pedro** (Logron˜o): Antonio Rus, Roberto Ruiz, Daniel Mosquera and Marta Casan˜as.
